# Comparative Evaluation of Surface Roughness and Hardness of 3D Printed Resins

**DOI:** 10.3390/ma15196822

**Published:** 2022-10-01

**Authors:** Yousif A. Al-Dulaijan, Leenah Alsulaimi, Reema Alotaibi, Areej Alboainain, Haidar Alalawi, Sami Alshehri, Soban Q. Khan, Mohammed Alsaloum, Hamad S. AlRumaih, Abdulkareem A. Alhumaidan, Mohammed M. Gad

**Affiliations:** 1 Department of Substitutive Dental Sciences, College of Dentistry, Imam Abdulrahman Bin Faisal University, P.O. Box 1982, Dammam 31441, Saudi Arabia; 2 Fellowship of Pediatric Dentistry Program, College of Dentistry, Imam Abdulrahman Bin Faisal University, P.O. Box 1982, Dammam 31441, Saudi Arabia; 3 College of Dentistry, Imam Abdulrahman Bin Faisal University, P.O. Box 1982, Dammam 31441, Saudi Arabia; 4 Department of Biomedical Dental Sciences, College of Dentistry, Imam Abdulrahman Bin Faisal University, P.O. Box 1982, Dammam 31441, Saudi Arabia; 5 Department of Dental Education, College of Dentistry, Imam Abdulrahman Bin Faisal University, P.O. Box 1982, Dammam 31441, Saudi Arabia; 6 Restorative and Prosthetic Dental Sciences Department, College of Dentistry, King Saud bin Abdulaziz University of Health Sciences, P.O. Box 3183, Riyadh 11426, Saudi Arabia; 7 King Abdullah International Medical Research Centre, Ministry of National Guard Health Affairs, P.O. Box 3183, Riyadh 11481, Saudi Arabia; 8 Department of Preventive Dental Sciences, College of Dentistry, Imam Abdulrahman Bin Faisal University, P.O. Box 1982, Dammam 31441, Saudi Arabia

**Keywords:** 3D printing, complete removable dental prosthesis, Vickers hardness, post-curing, build orientation

## Abstract

The effect of printing parameters on the surface characteristics of three-dimensional (3D)-printed denture base resins (DBRs) is neglected. Therefore, this study investigated the effect of printing orientation and post-curing time on the surface roughness and hardness. One conventional heat-polymerized (HP) resin and two 3D-printing resins (NextDent (ND) and ASIGA (AS)) were used to fabricate a total of 250-disc (10 × 2.5 mm) specimens. ND and AS specimens were printed with different orientations (0-, 45-, and 90-degree) and each orientation group was subjected to four post-curing times (30, 60, 90, 120 min). Printed specimens were thermo-cycled (10,000 cycles) followed by the measuring of surface roughness (Profilometer (Ra)) and hardness (a Vickers hardness (VH)). ANOVA and post hoc tests were used for data analysis (α = 0.05) at significant levels. AS and ND showed no significant changes in Ra when compared with HP (*p* ˃ 0.05), except the 45-degree orientation (AS/90 min and AS/120 min) significantly increased surface roughness (*p* ˂ 0.001). There was no significant difference in Ra with different orientations and post-curing time for both materials AS and ND (*p* ˃ 0.05). Compared with HP, 3D-printed DBRs showed low VH values (*p* ˂ 0.001). For AS, 90-degree orientation showed a significant decrease in VH at 60, 90, and 120 min when compared with 0- and 45-degree orientation (*p* ˂ 0.001), while ND showed no significant difference in VH with different printing orientations (*p* ˃ 0.05). The VH of AS and ND improved when increasing post-curing time to 120 min (*p* ˂ 0.001), and the printing orientations and post-curing time did not affect the Ra of 3D-printed DBRs.

## 1. Introduction

Poly methyl methacrylate (PMMA) has numerous advantages, making it the most clinically relevant material for removable prostheses fabrications [1]. However, some drawbacks were related to its poor surface properties [2]. Polished denture base surfaces are required to decrease microbial adhesion and discolorations with esthetic demands [3]. Moreover, denture base resins with abrasion resistance give the denture strength that is resistant to the mechanical brushing effect and the chewing of hard foods [4]. Denture base material with less abrasion-resistance ability may lead to increasing surface roughness and crack propagation at deep scratches in addition to the dimensional changes [5]. Therefore, denture base resins with adequate surface properties contribute to denture longevity with esthetic considerations even with evolved digital technologies for removable prostheses fabrications. 

Computer aided design–Computer aided manufacture (CAD-CAM) is applied technology for removable prosthesis production [6]. Two fabrication methods were used for the digital concept: subtractive (milling method) and additive (3D printing). The demand for 3D-printed denture materials has recently increased and this can explain the urge to conduct numerous research in this field. Due to some advantages of 3D printing, it received more attention than the milling technology [7,8]. These include cost-effectiveness, low material waste, no milling burs wearing, and easily reproductive prostheses with fine details. Three-dimensional printing also allows for more complex shapes, hence the design range is not limited [3,9]. Furthermore, using 3D printing aids in lesser appointments, shorter fabrication time, superior quality control, and improved strength and fit of the restoration [10]. Milled prostheses, on the other hand, have appropriate physicomechanical properties compared with 3D-printed prostheses [5,11]. 

Various variables must be kept under control during the process of 3D printing. Printed layer thickness, polymerization depth, amount of shrinkage, and volume and angle of the light source all influence the mechanical and physical properties [12]. It is critical to understand the various aspects that influence the qualities of the 3D printing result [12,13,14]. It is reported that the printing resin types, printing technology, and printing parameters are contributing factors affecting the properties of printed objects [15]. Amongst additive technologies, stereolithography (SLA) and digital-light processing (DLP) are most commonly used for removable prostheses built layer-by-layer using photocurable resin in a layering technique [3,5,16,17]. SLA 3D printing is a rapid method for manufacturing highly accurate and trueness complex shapes, mostly used to make resin-based objects. Furthermore, it can also be used to fabricate metal and ceramic objects with the need for additional de-binding and sintering processes after printing [18,19]. With this technology, the polymerization of the 3D-printed resin occurs in the XY coordinates by two mirrors that are used to direct a laser beam. On the other hand, DLP technology involves using a light-emitting diode (LED) light source that is projected as individual pixels onto the projection surface [20]. When comparing the two technologies, a localized polymerization of the 3D printing resin occurs when a laser beam travels through the layer surface in SLA, whereas, in the case of DLP, a light source is projected into the x/y space of the whole layer causing simultaneous curing [21]. 

In addition to printing technologies, printing parameters, (printing ordinations, printing layer thicknesses) and post-printing conditions (post-curing times and temperatures) were reported as factors affecting the properties of printed objects [15,20,22,23,24]. Printing orientation was reported as an influencing factor in the accuracy and mechanical properties of printed objects [22,23]. Unkovskiy et al., have investigated the printing orientation effects (horizontal, oblique, and vertically (0-, 45-, 90-degree)) and found that printing orientations affect the printed resin properties [24]. In 2020, Shim et al., investigated the effect of printing orientations on the characterization of 3D-printed resin and concluded the printing orientation had a considerable impact on the accuracy of printing, the flexural strength values, the surface roughness, and the Candida albicans reaction and deposition. As a result, to create items with adequate qualities, the printing orientation should be knowledgeably determined [25].

The post-curing process aims to enhance the printed prosthesis’ mechanical qualities. After printing, the photopolymerized resin is exposed to an extra curing step in an oven with an ultraviolet light source to ensure complete and equal polymerization of unreacted monomers, thereby improving the mechanical strength [9]. The extent of the post-curing process varies depending on the different photo-polymerized 3D-printed resins and different manufacturers’ specifications. It also differs significantly from 3D printing and post-curing equipment [26,27]. According to Aati’s findings [5], increasing the post-curing time up to 20 min will result in improving the physicomechanical properties of the 3D-printed DBR material, demonstrating equivalent performance to the traditional heat-cured control [28].

Although a few earlier studies have been undertaken investigating the effects of printing parameters on the mechanical properties of 3D-printed denture base resins (DBRs), no studies have evaluated the effect of post-curing time on the surface properties (surface roughness and hardness). Moreover, no studies have evaluated the combined effect. Thus, this study was conducted to assess the surface properties of printed specimens with different orientations and post-curing times. The null hypothesis was that orientation changes with different post-curing times do not affect the surface properties of the 3D-printed DBRs.

## 2. Materials and Methods

**Figure 1:**
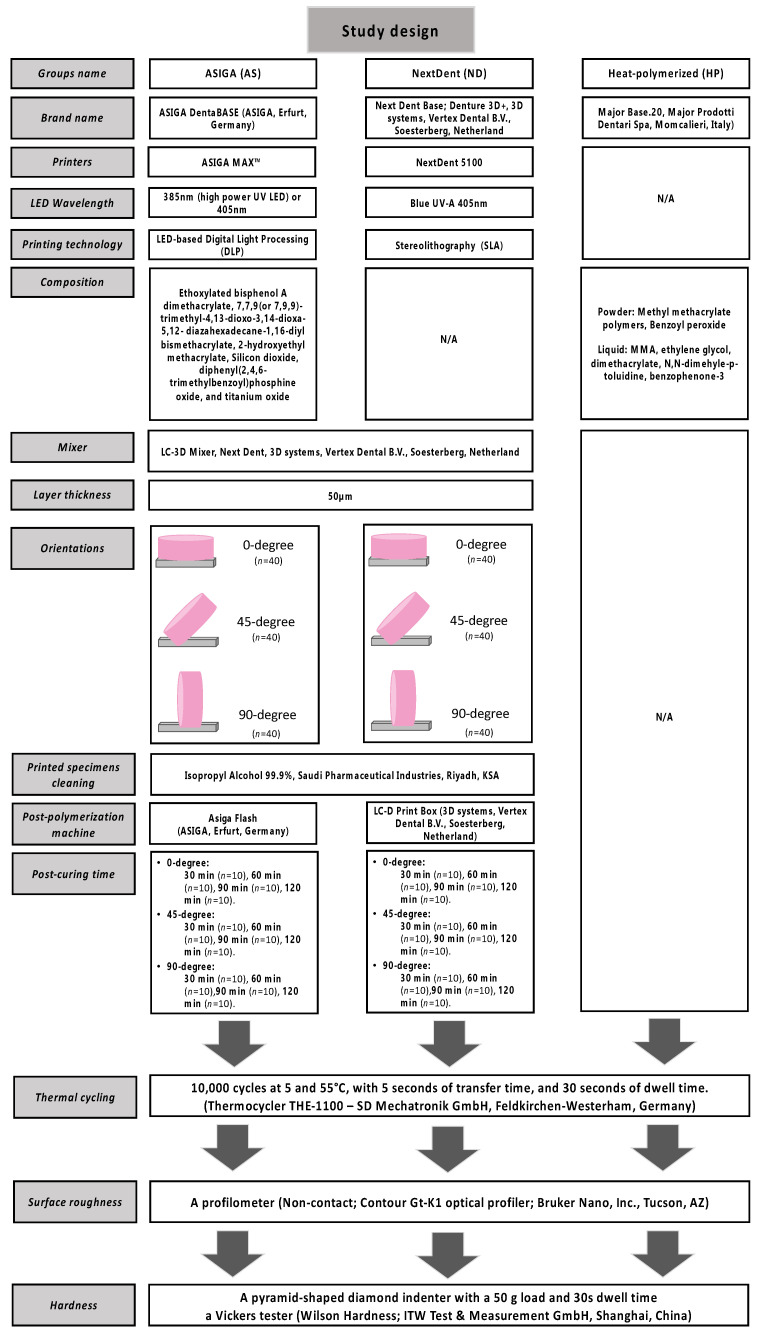
Study design and flowchart.

The sample size calculation equation was used based on previous studies [24,25] and revealed that a total of 250 specimens (10/HP control, 120/3D-printed resin, *n* = 10) were required (Figure 1). The disc-shaped specimen dimensions (10 × 2.5 mm) were used to evaluate hardness and surface roughness. For heat-polymerized (HP) denture base resin, specimens were prepared using a water bath conventional method as detailed in a previous study [29]. For 3D-printed resins, the disc specimen was designed virtually and then exported as standard tessellation language (STL) files to 3D printing systems (ASIGA and NextDent printers) [30]. The study design, including printing parameters and specifications, are listed in detailed in Figure 1.

**Figure 2:**
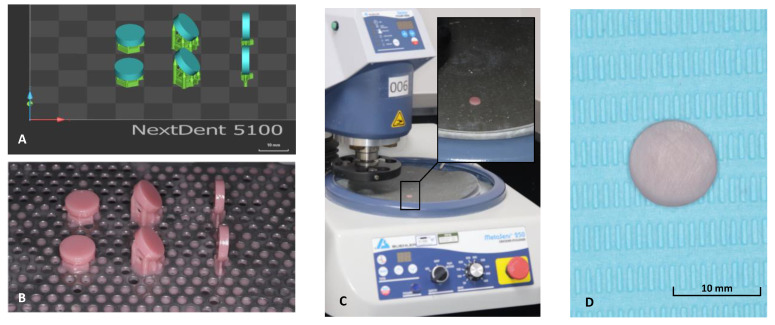
(**A**–**D**) Summarization of fabrication process 3D-printed samples. (**A**) Virtual design of the sample with printing orientation (0, 45, 90-degree), (**B**) printed samples before post-curing process, (**C**) polishing machine (AutoMet250, Buehler Ltd., Lake Bluff, IL, USA) with unpolished disc, and (**D**) polished disc.

Next, all the printing support structures were removed, followed by grounding the specimens with silicon carbide paper (800, 1500, and 2000 grit) and rinsing with water as recommended by Kwon et al., [31]. For specimen surface standardization, one investigator performed and completed the polishing procedures (Figure 2). The specimens’ dimensions and surface integrities were examined and then stored for 48 h in 37 °C distilled water [11]. This was followed by thermal cycling for 10,000 cycles in distilled water (Figure 1).

**Figure 3:**
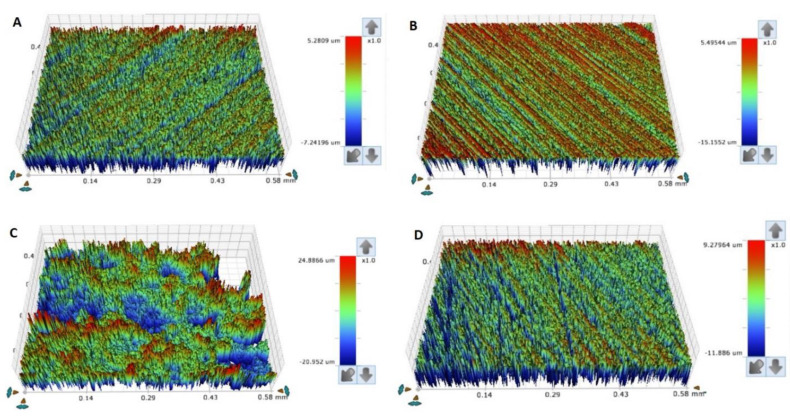
(**A**–**D**) Representative surface roughness images of scanned areas of HP and ASIGA groups at 120 min. (**A**) HP, (**B**) 0-degree, (**C**) 45-degree, (**D**) 90-degree.

**Figure 4:**
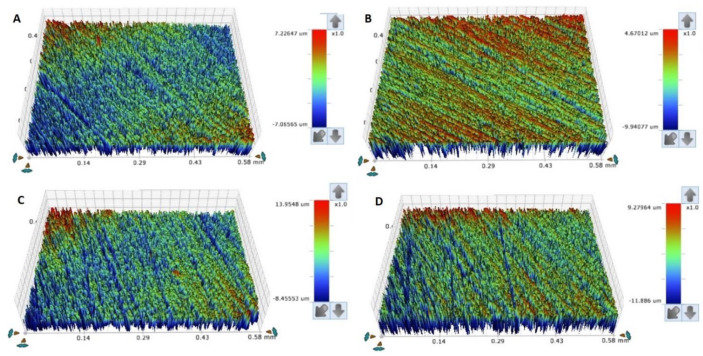
(**A**–**D**) Representative surface roughness images of scanned areas of HP and NextDent groups at 120 min. (**A**) HP, (**B**) 0-degree, (**C**) 45-degree, (**D**) 90-degree.

To evaluate the specimens’ surface roughness (surface roughness average, Ra), a profilometer (non-contact; Contour Gt-K1 optical profiler; Bruker Nano, Inc., Tucson, AZ, USA) was used, in which each specimen was mounted on the automated x-y stage. Five different points were scanned using scanning parameters as described by a previous study [32]. Following that, the capture images were used to calculate the averaged surface roughness (um) per specimen, Figure 3 and Figure 4 [3]. For the hardness value, a Vickers tester (Wilson Hardness; ITW Test and Measurement GmbH, Shanghai, China) was used with a pyramid-shaped diamond indenter used to indent each specimen with a 50 g load and 30 sec dwell-time at three different areas per specimen followed by average calculations. [5].

A Shapiro–Wilk test was used for normal data distribution and showed insignificant results, therefore parametric tests were used for data analysis. One-way ANOVA was used when only one factor was adopted (such as time or orientation) to study its effect on surface roughness and hardness. Two-way ANOVA was used for the combined effect. With significant findings, the post hoc Tukey HSD test was used for pairwise comparison. All *p*-values less than 0.05 were considered statistically significant.

## 3. Results

**Figure 5:**
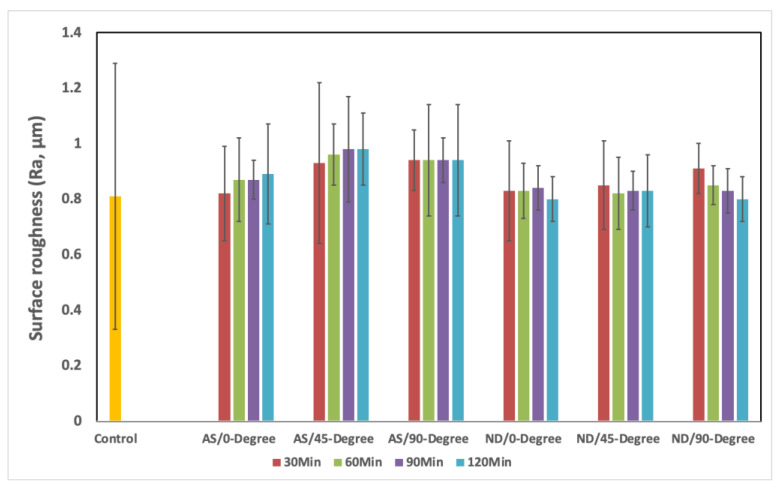
Mean and SD of surface roughness (Ra, µm) of all tested groups.

The mean and standard deviation (SD) of surface roughness of each group of orientation and post-curing time was summarized in Table 1 and Figure 5. The comparison of HP with AS and ND, separately for each factor (orientation and post-curing time) was analyzed and found most of the *p*-values were statistically significant. Only the significance was found due to variation in orientation degrees at 90 min and 120 min post-curing time (*p* = 0.013 and =0.028, respectively) (Table 1). The pairwise comparison provided that, at both 90 min and 120 min, the pair HP vs. 45-degree had a significant difference in means (*p* < 0.001). Similarly, in the comparison between HP with NextDent, when the orientation was at 90-degree, the variation due to post-curing time was found to be statistically significant (*p* = 0.032) (Table 1). In the pairwise comparison, the difference in means in HP vs. 30 min was statistically significant (*p* < 0.001). Furthermore, the combined effect of post-curing time and printing orientation on each material was analyzed using two-way ANOVA (Table 2). Results showed no significance regarding the combined effects per material (ASIGA; *p* = 0.768, and NextDent; *p* = 0.097).

**Figure 6:**
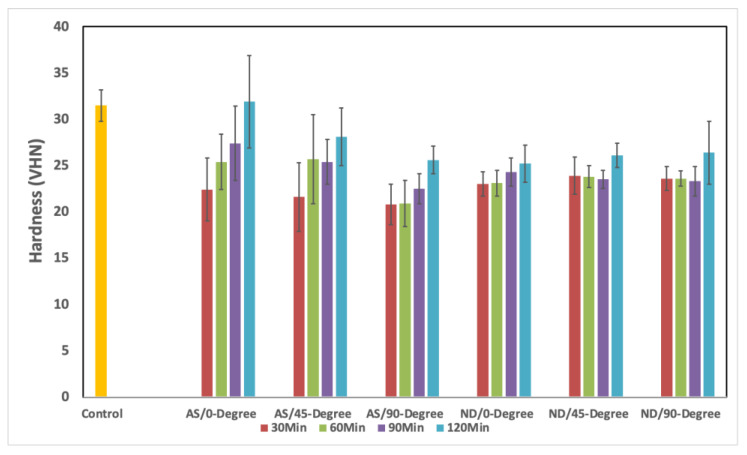
Mean and SD of hardness (VHN) of all tested groups.

The mean values, SD, and significance of hardness between groups are summarized in Table 3 and Figure 6. For AS, the variations were found statistically significant for all groups of degrees of orientation and post-curing time (Table 3). In the variation, because of the degrees of orientation, the pairs 45 vs. 90 at 30 min, 0 vs. 45 at 60 min, and 0 vs. 45 and 45 vs. 90 at 90 min and 120 min of post-curing time had statistically insignificant differences in means (*p* > 0.05). Similarly, in the variation due to the post-curing time, the pairs HP vs. 30, HP vs. 120, 30 vs. 60, 30 vs. 90, and 60 vs. 90 at 0-degree orientation had statistically insignificant differences in mean, while at 45-degree orientation, the pairs HP vs. 30, 60, and 90 and 30 vs. 120 had a significant difference in means (*p* < 0.05). Similarly, at 90-degree orientation, the pairs 30 vs. 60 and 90 and 60 vs. 90 had statistically insignificant differences in means. 

In the Next-Dent, the variation in mean due to the change in orientation was found statistically insignificant at 90 min and 120 min, with *p*-values of 0.946 and 0.415, respectively. While variation in mean due to change in post-curing time was found insignificant at 45-degree orientation (*p* = 0.086), the pairs 45 vs. 90 at 30 min and 90 min and 0 vs. 45 and 45 vs. 90 at 90 min and 120 min were found statistically insignificant. Furthermore, in the post-curing time, the pairs 30 vs. 90 and 90 vs. 120 at 0-degree orientation were statistically insignificant. At 45-degree, the pairs HP vs. 30, 60, 90, and 120 had significantly different means. At 90-degree, 30 vs. 60, 30 vs. 90, and 60 vs. 90 had statistically insignificant mean differences. The combined effect on the surface properties is shown in Table 4. For ASIGA, the combined effect showed no significant differences (*p* = 0.222), while a significant difference was found with NextDent material (*p* = 0.002).

## 4. Discussion

Aiming to improve 3D-printed DBR properties, modifications of printing parameters were suggested. The current study was conducted to investigate the effect of post-curing times and printing orientations on surface roughness and hardness. The results of this study showed that the printing orientations and post-curing times did not alter the surface roughness of 3D-printed DBRs, while hardness was affected. Thus, the null hypothesis was partially rejected. 

In the oral environment, the removable prostheses are subjected to thermal stress with daily use. To verify the clinical performances of the newly introduced technology, further studies evaluating the effects of several 3D printing resins in simulating oral conditions are necessary. Gale et al., reported that subjecting resin to 10,000 thermal cycles simulates 1 year of clinical denture use [3,33]. Moreover, the water sorption by resin affects the mechanical properties, hence absorbed water acts as a plasticizer. Meanwhile, increased temperature accelerated water uptake, thereby decreasing the mechanical properties [3].

Surface roughness is considered one of the most principal properties that might affect the longevity of removable properties, hence it increases microbial adhesion, which is ended by denture stomatitis [34]. Moreover, the rougher surfaces, more denture surface stains, and discoloration compromises denture esthetics [3,25,35]. The surface topography of additively manufacturing materials necessitates more investigations due to layering printing natures and technology [35,36]. Modifications of printing parameters and conditions affected the printed object surfaces. Additionally, the layering direction in relation to specimens’ surfaces might affect the surface properties [35]. It was stated that printing orientations have an impact on porosity and morphological features of printed objects [37,38,39]. According to the results of the present study, there is no change in Ra between HP, AS, and ND in terms of printing orientations, except for AS 45/90 and 45/120. This finding agrees with the prior investigation [3] that compared the surface roughness of 3D-printed resin with HP before and after thermal cycling and that there was no significance after thermal cycling. However, there is no significant change but variations in values that show an increase in surface values with 45- and 90-degree when compared with 0-degree. These variations may be attributed to printing layers with steps between adjacent layers, while 0-degree represented one layer in the horizontal plane per Shim et al., who reported that 0-degree showed the lowest Ra value than 45- and 90-degree [25].

Regarding the post-curing time effect, results revealed no change in surface roughness as the post-curing time increased. As per tested material, there was no significant effect with combined printing orientations and post-curing time, which confirmed that both factors have no remarkable effects on the surface roughness of DBRs. The fact behind the post-curing time is based on the degree of monomer conversion, which finally improved the printed object properties, as reported by Lee et al., [40]. Lee et al., [40] confirmed that post-curing time increased the degree of monomer conversion. However, this fact was not significantly proven in the present study, but a slight decrease in Ra with the ND/45 and ND/90 groups as the post-curing time increased insignificantly. Moreover, the post-curing method did not affect the roughness, in agreement with Li et al., [28], who found that various post-curing settings did not significantly change the Ra values [28].

The hardness of the specimen surface is an indicator of abrasion resistance and reflects the material’s surface strength in case of low hardness, more scratches, damage to the resin surface, and dimensional changes that may occur with mechanical denture brushing or chewing hard food substances [3,5]. The 3D-printed denture bases’ average hardness ranges from 30.17 to 34.62 VHN and their average surface roughness is between 0.12 and 0.22 μm [3]. As the hardness decreased, the roughness increased in inverse relations [3,25,28], making the correlations between the two surfaces’ properties reasonable to be selected in the present study. The 3D-printed resins showed low hardness compared with the control, agreeing with previous studies [3,41]. After thermal cycling, the hardness value may decrease due to water sorption and alteration of material and printing layers’ composition and when thermally stressed. Based on a previous comparative study of non-thermal cycled materials [41], the hardness values of 3D-printed materials were the lowest compared with HP and subtractive manufacturing materials. In this study, comparable results for thermally and non-thermally cycled 3D-printed resins were reported.

In the present study, the hardness of both 3D-printed resins did not change significantly. This may be attributed to the behaviors of the resins in terms of water sorption and thermal stress effects [3]. However, hardness was increased with increasing post-curing time. In agreement with Lee et al.,’s finding [40], the increase in hardness value in the group with 120 post-curing times may be attributed to the polymerization rate and the degree of conversion. Based on the results of the present study, a direct relation between the hardness and the post-curing time was reported as the hardness was significantly increased when the post-curing time increased [5]. Furthermore, different 3D-printed resins have been post-cured for up to 120 min, which considerably improved the hardness values, making them comparable with the HP group. It was confirmed that longer post-curing times result in a better degree of conversion, leading to a lower residual monomer content, therefore increasing hardness [5,26,42].

The selection of denture base materials depended mainly on surface characteristics. Discoloration, water absorption, microbial adhesion, and even oral hygiene can be impacted by surface characteristics such as surface topography and roughness [25]. Therefore, the surface properties of 3D-printed DBRs should be assessed for clinical applicability. The 3D-printed DBRs displayed lower surface properties in comparison with conventional heat-polymerized. Although this study modified some printing parameters to improve the surface properties, only the post-curing time improved the hardness, while printing orientations had no effects. Therefore, increasing the post-curing time to 90–120 min is recommended. The present study’s findings reflected the effect of the post-curing effect on the surface properties of DBRs with a more reliable method that could improve the properties of dentures with real configurations. Hence, the whole denture will be subjected to long polymerization, resulting in the improvement of physical–mechanical properties through the inherent degree of conversion characteristics. Printing orientation depended mainly on the load and printing layer direction which could be standardized with bar-shaped specimens. Denture configuration is different, and within the same denture all orientations might have occurred according to the curvature of the denture surface. Therefore, it is more applicable if future research focuses on other factors in addition to post-curing time, resin composition modification [18], printed resin reinforcement [43], and/or post-curing methods with different curing units [28]. Additionally, further investigations on the accuracy of 3D printed resins with the aforementioned parameters are recommended [44] 

Using different denture base resins with the combination of printing parameters and thermal cycling effects are sources of strength of the present study. Nevertheless, some limitations include the tested bar-shaped specimens that differ from real denture configurations and the absence of oral environments and mechanical stresses from the musculature system, in addition to the absence of wear resistance evaluation that is considered one of the related surface properties. Based on the roughness results of this study, future research is required to develop and examine the effect of different polishing protocols on the surface properties of 3D-printed DBRs. Additionally, further investigations in conditions simulating an oral environment are required to evaluate other printing technologies and parameters. In addition, combined effects of printing parameters that affect the properties in vivo are required.

## 5. Conclusions

Printing parameters (orientation and post-curing time) for two different 3D-printed resins were investigated in the present study. 

In both 3D-printed DBRs, there is no change and no impact on the surface roughness in terms of printing orientations and post-curing time effects. 

On the other hand, the printing orientation does not affect the hardness, while the post-curing time does affect the hardness. Increasing post-curing time by 120 min improved the surface hardness. 

The 3D-printed denture base resins that were subjected to 120 min of post-curing times exhibited comparable hardness to heat-polymerized DBRs. 

Finally, an increase in the post-curing time improved the hardness of the 3D-printed denture base material, while the printing orientation did not.

## Figures and Tables

**Table 1:** Mean, SD, and significance of surface roughness (µm) test between groups.

Materials	Orientation	Post-Curing Time				HP	*p*-Value
		30 min	60 min	90 min	120 min		
**HP**		0.81 ± 0.048	0.81 ± 0.04	0.81 ± 0.048 ^a^	0.81 ± 0.048 ^a^		
**ASIGA**	0	0.82 ± 0.17	0.87 ± 0.15	0.87 ± 0.07	0.89 ± 0.18	0.81 ± 0.048	0.603
	45	0.93 ± 0.29	0.96 ± 0.11	0.98 ± 0.19 ^a^	0.98 ± 0.13 ^a^	0.81 ± 0.048	0.929
	90	0.94 ± 0.11	0.94 ± 0.20	0.94 ± 0.08	0.94 ± 0.20	0.81 ± 0.048	0.56
	*p*-value	0.117	0.061	0.013	0.028		
**HP**		0.81 ± 0.048	0.81 ± 0.048	0.81 ± 0.048	0.81 ± 0.048		
**NextDent**	0	0.83 ± 0.18	0.83 ± 0.10	0.84 ± 0.08	0.80 ± 0.08	0.81 ± 0.048	0.184
	45	0.85 ± 0.16	0.82 ± 0.13	0.83 ± 0.07	0.83 ± 0.13	0.81 ± 0.048	0.082
	90	0.91 ± 0.09 ^A^	0.85 ± 0.07	0.83 ± 0.08	0.80 ± 0.08	0.81 ± 0.048 ^A^	0.032 *
	*p*-value	0.447	0.103	0.350	0.331		

* Statistically significant at 0.05 level of significance. Same small letter indicated significance between pairs per column, while same capital letter indicated significance between pairs per raw (*p* ˃ 0.05). No letters added vertically or horizontally when *p* values were insignificant.

**Table 2:** Two-way ANOVA for combine effect of orientation and post-curing time on the surface roughness of each material.

Materials	Source	Type III Sum of Squares	*df*	Mean Square	*F*-Value	*p*-Value
**ASIGA**	Post-curing time	0.010	3	0.003	0.124	0.946
	Orientation	0.375	2	0.187	7.011	0.001 *
	Post-curing time * orientation	0.089	6	0.015	0.552	0.768
	Error	3.128	117	0.027		
	Total	111.792	130			
	Corrected Total	3.720	129			
**NextDent**	Post-curing time	0.128	3	0.043	3.667	0.014 *
	Prientation	0.102	2	0.051	4.381	0.015 *
	Post-curing time * orientation	0.128	6	0.021	1.842	0.097
	Error	1.360	117	0.012		
	Total	87.108	130			
	Corrected Total	1.718	129			

* Statistically significant at 0.05 level of significance.

**Table 3:** Mean, SD, and significance of hardness (VHN) test between groups.

Materials	Orientation	Post-Curing Time				HP	*p*-Value
		30 min	60 min	90 min	120 min		
**HP**		31.48 ± 1.7	31.48 ± 1.7	31.48 ± 1.7	31.48 ± 1.7		
**ASIGA**	0	26.4 ± 3.4 ^C,D^	25.3 ± 3.0 ^a,C,E^	27.4 ± 4.0 ^a,A,D,E^	31.9 ± 5.0 ^a,B^	31.48 ± 1.7 ^A,B^	0.002 *
	45	21.6 ± 3.7 ^a,B,C^	25.7 ± 4.8 ^a,B,D,E^	25.4 ± 2.4^a,b,C,D,F^	28.1 ± 3.1 ^a,b,A,E,F^	31.48 ± 1.7 ^A^	0.003 *
	90	20.8 ± 2.2 ^a,A,B^	20.9 ± 2.5 ^A,C^	22.5 ± 1.6 ^b,B,C^	25.6 ± 1.5 ^b^	31.48 ± 1.7	0.000 *
	*p*-value	0.001 *	0.009 *	0.003 *	0.002 *		
**HP**		31.48 ± 1.7	31.48 ± 1.7	31.48 ± 1.7	31.48 ± 1.7		
**NextDent**	0	23.0 ± 1.3 ^A^	23.1 ± 1.4 ^A^	24.3 ± 1.5 ^a,B^	25.2 ± 2.0 ^a,B^	31.48 ± 1.7	0.000 *
	45	23.9 ± 2.0 ^a,A,B,C^	23.8 ± 1.2 ^a,A,D,E^	23.5 ± 1.0 ^a,b,B,D,F^	26.1 ± 1.3 ^a,b,C,E,F^	31.48 ± 1.7	0.086
	90	23.6 ± 1.3 ^a,A,B^	23.6 ± 0.8 ^a,A,C^	23.3 ± 1.6 ^a,b,B,C^	26.4 ± 3.4 ^b^	31.48 ± 1.7	0.006 *
	*p*-value	0.000 *	0.000 *	0.946	0.415		

* Statistically significant at 0.05 level of significance. Small alphabets showed insignificant difference between the pairs within the same column, while capital alphabets showed insignificant difference between the pairs within the same raw.

**Table 4:** Two-way ANOVA for combined effect of orientation and post-curing time on the hardness of each material.

Materials	Source	Type III Sum of Squares	*df*	Mean Square	*F*-Value	*p*-Value
**ASIGA**	Post-curing time	532.896	3	177.632	17.472	0.000 *
	Orientation	617.057	3	205.686	20.231	0.000 *
	Post-curing time * orientation	85.198	6	14.200	1.397	0.222
	Error	1189.494	117	10.167		
	Total	88,005.939	130			
	Corrected Total	2732.322	129			
**NextDent**	Post-curing time	167.209	3	55.736	19.356	0.000 *
	Orientation	345.612	3	115.204	40.007	0.000 *
	Post-curing time * orientation	66.674	6	11.112	3.859	0.002 *
	Error	336.910	117	2.880		
	Total	77,150.437	130			
	Corrected Total	1230.048	129			

* Statistically significant at 0.05 level of significance.

## Data Availability

Not applicable.

## Abbreviations

3D	Three-dimensional
ANOVA	Analysis of variance
AS	ASIGA
CAD-CAM	Computer-aided design-Computer-aided manufacturing
DBRs	Denture base resins
DLP	Digital light processing
HP	Heat polymerized
LED	Light-emitting diode
µm	Micrometer
ND	NextDent
PMMA	Poly methyl methacrylate
Ra	Surface roughness average
SD	Standard deviation
SLA	Stereolithography
STL	Standard tessellation language
VH	Vickers hardness
VHN	Vickers hardness number
